# Paying for clinical AI: The bill is not the cost

**DOI:** 10.1371/journal.pdig.0001814

**Published:** 2026-11-02

**Authors:** Vishva Natarajan, Caleb Stewart, Jennifer Hong, Saeed Hassanpour

**Affiliations:** 1 Geisel School of Medicine at Dartmouth, Hanover, New Hampshire, United States of America; 2 Department of Neurosurgery, Dartmouth-Hitchcock Medical Center, Lebanon, New Hampshire, United States of America; 3 Departments of Biomedical Data Science, Computer Science, and Epidemiology, Dartmouth College, Hanover, New Hampshire, United States of America; Australian Institute of Health Innovation, AUSTRALIA

## Abstract

Clinical AI is increasingly integrated into electronic health record workflows, but its cost is difficult to define. Health systems may pay through usage-based services, subscription or enterprise licenses, local hosting, or combinations of these approaches. Usage-based pricing introduces a distinct problem because billing units such as tokens or billable sessions are vendor-defined and may vary across data types, languages, and over time. Fixed licensing agreements can make vendor charges more predictable, while locally hosted AI models shift spending toward infrastructure and personnel. Regardless of the payment structure, the full resources required to use and maintain AI, including engineering, validation, oversight, and rights to clinical data, are difficult to capture. As the complexity of multimodal and agentic systems increases, so will that of the payment structures. Health systems will need to proactively develop ways to accurately measure the cost of AI use, evaluate its clinical value, and negotiate agreements with AI vendors that allow for regular reassessment of prices and terms. Doing so will help ensure that AI does not become yet another driver of healthcare spending detached from value delivered.

## How health systems pay for AI

Generative and multimodal AI systems are moving into electronic health record workflows, supporting point-of-care reasoning [1], imaging review [2], and multi-step tool use [3], through interfaces ranging from ambient documentation systems to natural-language assistants in which clinicians prompt a large language model (LLM) that can call on multiple underlying AI tools. Health systems can pay for clinical AI in three ways. Usage-based services charge according to consumption, often through vendor-defined units such as tokens for generative models, or per study, inference, session, or API call for other AI systems [4]. Alternatively, subscription and enterprise licenses provide access for a fixed or recurring fee, so charges are less directly tied to each AI interaction.

A third option is local deployment, which follows the idea of “AI Sovereignty,” where a healthcare system hosts AI models on its own computational infrastructure rather than paying for requests to remote servers. Local deployment shifts spending from fees toward GPUs and servers, data storage, networking, power and cooling, and the personnel required to maintain them. These investments can demand substantial up-front capital, placing local deployment out of reach for many smaller or safety-net health systems. Health systems may also lack the resources to develop and validate in-house AI models themselves, making locally deployable commercial or open-weight AI models important alternatives to vendor-hosted services [5].

In practice, healthcare systems will likely use mixed payment models for clinical AI. As clinical AI shifts toward multimodal and agentic systems that combine multiple tools within a single task, health systems may host some models locally, license others, and pay per use for external services they cannot feasibly develop themselves. The same product may also span multiple pricing models. Dragon Copilot (DAX), for example, is available through subscription, pay-as-you-go, and hybrid arrangements with extra charges for usage beyond individual allowances [6]. The picture is further complicated by the potential role of insurance reimbursement in shaping the choice among models. Just as payer formularies influence which medications are financially accessible to patients, coverage and payment policies may similarly shape which AI investments are viable for health systems, even when health systems ultimately choose how the technology is purchased or deployed [7,8].

## When the billing unit changes

Usage-based pricing introduces a problem that is largely avoided by enterprise licensing and local deployment: relying on a vendor-defined unit for billing. Usage-metered LLMs frequently employ token billing, which resembles an electricity bill in that small charges accumulate across encounters, clinicians, and health systems. In one cost simulation, a clinical note was charged as 450 tokens and an extraction question as 10 tokens, but unlike a kilowatt-hour, a token is not standardized [9]. The same Spanish text required 1.57 times the English token count under one OpenAI tokenizer and 1.33 times under another [10], highlighting both the instability of tokenization and the potential for language premiums to increase AI costs for health systems serving non-English-speaking patient populations [11,12]. Images are likewise assigned input-token counts according to vendor-specific rules [13] that are highly variable.

Moving away from tokens does not eliminate the problem of transparency for AI charges. While DAX charges are calculated as $0.25 per AI-Assisted Session, this alternate billing unit is also ambiguous, with a single ambulatory encounter counting as one session but an inpatient encounter potentially counting as several [6]. As agentic systems incorporate multiple models and tools, vendors may similarly determine how those underlying calls are grouped into billable sessions or other units, making charges difficult to compare across vendors and over time. The problem may become more consequential as a single clinical task often draws on text, imaging, reasoning, and other model calls, making AI-related spending increasingly difficult for health systems to track.

## Beyond the invoice

Whether the AI bill is token-based, session-based, or a fixed enterprise license, it does not necessarily capture everything the health system is paying in exchange for services. At one academic health system, DAX was made available to more than 2,400 clinicians with no apparent added cost per user, yet deployment was supported by a ten-person team for technical implementation, training, user support, and ongoing troubleshooting [14]. Payment may also be nonmonetary. Where access agreements between hospitals and AI vendors grant rights to clinical data for commercial use, a lower bill may mask the value of robust training datasets granted by the health system to the vendor to train and market new models, without reciprocal profit-sharing with the hospital [15].

Locally hosted AI shifts costs more directly onto the health system, with personnel salaries exceeding $100,000 per year and GPU servers costing more than $200,000 in one instance [16]. These visible infrastructure costs are only one component of implementation, because workflow design can also materially affect AI spending. In one study, batching 50 clinical extraction questions into a single prompt reduced costs by up to seventeen-fold compared with submitting them separately [9], showing how small workflow changes can materially affect spending at scale. The engineering required to identify, implement, and maintain these efficiencies, along with any supporting hardware and infrastructure, is itself part of the cost of clinical AI. Additionally, value attributed to AI may partly reflect changes to hospital workflows made alongside its implementation. In one cardiothoracic operating suite, image-based AI was paired with turnover reviews, anesthesia workflow review, and surgeon notifications and was associated with a 7% increase in monthly surgical case volume, although the study could not separate the contribution of the AI from the accompanying workflow changes [17].

## Governing AI spending

As the complexity of clinical applications for AI grows, it is paramount that the cost of clinical AI is transparent and readily auditable so that AI does not become yet another uncontrolled source of healthcare spending that ultimately falls on patients. Three principles ought to guide AI procurement and implementation [18].

First, account for the full cost, not only the vendor bill. Health systems should precisely calculate all resources committed to AI, including vendor payments, infrastructure, engineering, validation, monitoring, and oversight, as well as any clinical data exchanged or rights granted for their future use [19]. These costs should be reassessed regularly after implementing new AI-driven workflows as usage and infrastructure requirements evolve. Health systems can then budget and compare AI systems based on their total resource requirements rather than the price that appears on the vendor invoice.

Second, evaluate AI using both task-specific performance measures and downstream clinical or operational outcomes. Tokens, sessions, and model calls describe how AI is consumed and billed, but not how much a health system ultimately benefits from using it. Measures such as diagnostic accuracy, clinical note quality, and administrative accuracy show whether an AI system performs its intended function, while clinician workload, turnaround time, and patient throughput show whether that performance translates into better care delivery [17,18,20]. Renewal of existing AI or adoption of newer systems should depend on whether the AI-enabled workflow improves care delivery compared with the existing workflow, including a human-only one where relevant [18]. Otherwise, health systems risk optimizing spending around what vendors can easily measure and bill rather than what actually improves care for patients.

Third, preserve the ability to change course. Usage-based agreements should state how vendor billing units map to institution-defined budgeting units and require disclosure when prices or unit definitions change. Health systems should also design and implement AI workflows so that validated tools can be replaced with clinically comparable alternatives without rebuilding surrounding workflows. Otherwise, technical and contractual switching costs may leave a health system tethered to one vendor even when a cheaper or better alternative becomes available.

## Conclusion

As clinical AI adoption expands, health systems should distinguish how AI is priced from what it ultimately costs and the clinical value it delivers. Usage-based, subscription, local, and hybrid arrangements distribute costs differently, while vendor-defined billing units can introduce additional uncertainty. Health systems should therefore account for the full resources committed for AI implementation, assess performance against relevant clinical workflows, and preserve the technical and contractual ability to switch vendors when prices or terms become unfavorable. Establishing these conditions is paramount so that AI does not become yet another driver of healthcare spending that is detached from the value delivered.

## References

[1] Shah NH, Ambers N, Pandya A, et al. Adoption and use of LLMs at an academic medical center. 2026;2602.00074.

[2] Han T, Lee JH, Jeong WK, Shin J, Roh YH, Kim MJ. Relationship between resource utilization and diagnostic accuracy of large language models for efficient multimodal reasoning in radiologic image interpretation. Eur J Radiol. 2026;196:112677. doi: 10.1016/j.ejrad.2026.112677 41576425

[3] Liu Y, Carrero ZI, Jiang X, Ferber D, Wölflein G, Zhang L, et al. Benchmarking large language model-based agent systems for clinical decision tasks. NPJ Digit Med. 2026;9(1):259. doi: 10.1038/s41746-026-02443-6 41708802 PMC13031301

[4] Kirchhoff J, Berns F, Schieder C, Schobel J. Pricing models for diagnostic AI based on qualitative insights from healthcare decision makers. NPJ Digit Med. 2026;9(1):231. doi: 10.1038/s41746-026-02501-z 41792432 PMC12996594

[5] Proff AK, Salam B, Hayawi M, Kravchenko D, Mesropyan N, Aziz-Safaie T, et al. Simplifying radiology reports with large language models: Privacy-compliant open- versus closed-weight models. Eur Radiol. 2026;36(7):6097–106. doi: 10.1007/s00330-026-12329-6 41677855 PMC13282196

[6] Microsoft. Dragon Copilot licensing guidance. Microsoft; 2026 [cited 2026 Aug 27]. Available from: https://www.microsoft.com/licensing/guidance/Dragon-Copilot

[7] Razavian N, Batchu-Green P, Chowdhry V, Elemento O, Rajpurkar P, Saria S, et al. Catalyzing health AI by fixing payment systems. NEJM AI. 2025;2(12):10.1056/aipc2500871. doi: 10.1056/aipc2500871 41695240 PMC12900248

[8] Mahajan A, Powell D. Generalist medical AI reimbursement challenges and opportunities. NPJ Digit Med. 2025;8(1):125. doi: 10.1038/s41746-025-01521-5 40011639 PMC11865540

[9] Klang E, Apakama D, Abbott EE, Vaid A, Lampert J, Sakhuja A, et al. A strategy for cost-effective large language model use at health system-scale. NPJ Digit Med. 2024;7(1):320. doi: 10.1038/s41746-024-01315-1 39558090 PMC11574261

[10] Solatorio AV, Vicente GS, Krambeck H, Dupriez O. Double jeopardy and climate impact in the use of large language models: Socio-economic disparities and reduced utility for non-English speakers. 2024. doi: 10.48550/arXiv.2410.10665

[11] Roy A, Roy P, Patel H. Measuring the tokenization premium: A cost audit for underserved language communities. 2608.09046.

[12] Petrov A, La Malfa E, Torr PHS, Bibi A. Language model tokenizers introduce unfairness between languages. In: Advances in neural information processing systems 36 (NeurIPS 2023). 2023.

[13] Bhalgami R. Pixels for programs? A cross-provider case study of input-token accounting for source code as text and images. arXiv 2026;2607.21672.

[14] Wright AP, Nall CK, Franklin JJH, Horst SN, Kumah-Crystal YA, Wright AT, et al. Enterprise-wide simultaneous deployment of ambient scribe technology: Lessons learned from an academic health system. J Am Med Inform Assoc. 2026;33(2):457–61. doi: 10.1093/jamia/ocaf186 41175896 PMC12844588

[15] Wilson J, Herron D, Nachev P, McNally N, Williams B, Rees G. The value of data: Applying a public value model to the english national health service. J Med Internet Res. 2020;22(3):e15816. doi: 10.2196/15816 32217501 PMC7148544

[16] Burns ML, Chen S-Y, Tsai C-A, Vandervest J, Pandian B, Nong P, et al. Generative AI costs in large healthcare systems, an example in revenue cycle. NPJ Digit Med. 2025;8(1):579. doi: 10.1038/s41746-025-01971-x 41028226 PMC12485018

[17] Nguyen N-AA, Lee G, Sossong S, Machnik JV, Pletcher S, Schwartz R. Implementation of image-based artificial intelligence is associated with increased case volume in a high-acuity, 15-room cardiothoracic operating suite at a tertiary academic hospital. J Imaging. 2026;12(7):283. doi: 10.3390/jimaging12070283 42506129 PMC13412611

[18] Khan SD, Hoodbhoy Z, Raja MHR, Kim JY, Hogg HDJ, Manji AAA, et al. Frameworks for procurement, integration, monitoring, and evaluation of artificial intelligence tools in clinical settings: A systematic review. PLOS Digit Health. 2024;3(5):e0000514. doi: 10.1371/journal.pdig.0000514 38809946 PMC11135672

[19] Cole CL, Sengupta S, Rossetti Née Collins S, Vawdrey DK, Halaas M, Maddox TM, et al. Ten principles for data sharing and commercialization. J Am Med Inform Assoc. 2021;28(3):646–9. doi: 10.1093/jamia/ocaa260 33186458 PMC7936510

[20] Bedi S, Cui H, Fuentes M, et al. MedHELM: Holistic evaluation of large language models for medical tasks. 2025. doi: 10.48550/arXiv.2505.23802 PMC13267972 41559415

